# Molecular Epidemiology of the *bla*_CTX-M_ Gene in *Escherichia coli* from a Pig Farm: Antimicrobial Resistance Profiles, Genetic Background, and Its Horizontal Transfer and Environmental Dissemination

**DOI:** 10.3390/microorganisms14051007

**Published:** 2026-04-29

**Authors:** Ri-Han Jiang, Zi-Kui Liu, Bing Han, Dan-Ni Liao, Ji-Yun Li, Yong Wu

**Affiliations:** College of Veterinary Medicine, Hunan Agricultural University, 1 Nongda Rd., District Furong, Changsha 410128, China; jrh1028@126.com (R.-H.J.);

**Keywords:** *Escherichia coli*, antimicrobial resistance, β-lactam resistance, mobile plasmids, horizontal gene transfer, pig farm

## Abstract

This study investigated the epidemiology, antimicrobial resistance, and transmission risks of β-lactamase, cefotaxime-hydrolyzing, Munich (*bla*_CTX-M_)-positive *Escherichia coli* (CTX-M-EC) in large-scale pig farms in Jiangxi Province (China). In total, 278 samples (manure, wastewater, drinking water, and flies) were collected. CTX-M-EC strains were isolated and analyzed using antimicrobial susceptibility testing, resistance gene profiling, multilocus sequence typing, and genetic environment analysis with gene transfer assessed by transduction experiments. Twenty-seven CTX-M-EC strains (9.71%) were isolated, all exhibiting multi-drug resistance with 100% resistance to cefotaxime, ciprofloxacin, and tetracycline, and >90% resistance to ceftazidime, florfenicol, and trimethoprim-sulfamethoxazole. Four *bla*_CTX-M_ subtypes were identified. *bla*_CTX-M-55_ was the predominant subtype (70.37%) and was distributed across diverse sequence types and serotypes. Each strain harbored multiple antibiotic resistance genes, plasmids, and virulence genes. Mobile elements such as IS*Ecp1* and IS*26* were detected surrounding the *bla*_CTX-M_ gene, and 96.29% of strains successfully transferred the *bla*_CTX-M_ gene via transduction. Clones highly homologous to pig manure strains were detected in flies and sewage, suggesting that this resistance gene can spread between animals, the environment, and vectors. These findings highlight the high transmission risk of *bla*_CTX-M_ and underscore the need for rational antibiotic use, waste management, and vector control within a One Health framework.

## 1. Introduction

Antimicrobial resistance (AMR) has been recognized as one of the most serious threats to global public health in the 21st century. The World Health Organization (WHO) has listed AMR among the top ten global health threats [1]. Moreover, the number of deaths directly attributable to AMR is projected to reach 1.91 million worldwide by 2050 [2]. Currently, China faces a similarly severe situation regarding bacterial resistance: according to the 2024 report of the CHINET China Bacterial Resistance Surveillance Network, the antimicrobial resistance of clinically isolated bacteria remains very high in major regions of China [3]. *Escherichia coli* (*E. coli*), a common opportunistic intestinal pathogen both in humans and animals, has become one of the core vectors for antimicrobial resistance dissemination [4], frequently causing various diseases, including diarrhea and septicemia in piglets as well as mastitis and endometritis in sows [5,6,7]. Third- and fourth-generation cephalosporins are commonly used for treatment during farming operations. However, bacteria producing extended-spectrum beta-lactamases (ESBLs) are also proliferating [8].

In Gram-negative bacteria, ESBL production represents the primary resistance mechanism against third-generation cephalosporins and other beta-lactam antibiotics. Among these enzymes, the *bla*_CTX-M_ genotype was first identified in Germany in 1989, having become the global predominant ESBL type [9]. Epidemiological data from different continents indicate significant geographical differences in CTX-M prevalence: in Europe, *bla*_CTX-M-1_ remains dominant [10]; in North America, *bla*_CTX-M-15_ is the predominant subtype, often associated with the epidemic clone ST131 [11]; in South America, *bla*_CTX-M-55_ is more common [12]; in South Africa, *bla*_CTX-M-15_ is the major epidemic genotype, with a higher prevalence in animal- than in human-derived samples [13]; in Asia, the dominant epidemic genotypes are *bla*_CTX-M-14_ and *bla*_CTX-M-55_ [10]. In China, *bla*_CTX-M-14_, *bla*_CTX-M-15_, and *bla*_CTX-M-55_ are common variants [14], among which *bla*_CTX-M-55_ is predominant in animal sources, whereas *bla*_CTX-M-14_ is more common in the human population [15,16].

CTX-M-type ESBL dissemination primarily relies on two core mechanisms [17]. First, horizontal gene transfer, involving genes typically located on plasmids that often carry multiple antibiotic resistance genes (ARGs) and can spread horizontally between bacterial species, thereby accelerating the proliferation of multi-drug resistance (MDR) strains [10]. Second, clonal dissemination, driven by highly adaptable clonal strains. ST131 is widely recognized as the most important clonal vector for the spread of CTX-M-type ESBLs and the primary carrier of *bla*_CTX-M-15_, but it can also harbor various other subtypes such as *bla*_CTX-M-14_ and *bla*_CTX-M-27_ [18]. ST38 is the second most common sequence type, capable of carrying multiple *bla*_CTX-M_ variants, including *bla*_CTX-M-14_ and *bla*_CTX-M-15_ [19]. ST410 is an emerging “One-Health” high-risk clone with significant potential to acquire carbapenemases and evolve into an MDR phenotype. Furthermore, ST410 has been widely detected in human, animal, and environmental sources [20].

The rapid spread of CTX-M-type extended-spectrum β-lactamases (ESBLs) in animal sources not only poses a serious threat to the healthy development of the livestock industry and increases production costs, but may also transmit resistance risks to human society through the food chain and environmental media, thereby constituting a major public health threat [21]. Therefore, this study involved the collection of pig manure and environmental samples from a large-scale pig farm in Jiangxi Province, China. On the basis of *bla*_CTX-M_ gene detection, epidemiological analysis, and gene transfer experiments of *E. coli* isolates, this study aimed to elucidate the prevalence, antimicrobial resistance characteristics, and potential transmission risks of *bla*_CTX-M_-positive *E. coli* (CTX-M-EC) on this pig farm.

## 2. Materials and Methods

In 2022, 278 samples (pig manure, wastewater, drinking water, and flies) were collected from a pig farm in Pingxiang City, Jiangxi Province, China. The samples were inoculated in Lysogeny Broth (Beijing Luqiao Technology Co., Ltd., Beijing, China) and incubated in a constant-temperature air shaker (Eppendorf China Ltd., Shanghai, China) at 37 °C for 16–18 h for enrichment. The bacterial cultures were then streaked onto MacConkey agar (Beijing Luqiao Technology Co., Ltd.) supplemented with 2 mg/L cefotaxime (Shanghai Aladdin Biochemical Technology Co., Ltd., Shanghai, China) for isolation and purification of ESBL-producing *E. coli*. Strains carrying the *bla*_CTX-M_ gene were screened by polymerase chain reaction (PCR) and agarose gel electrophoresis [22]. After species identification as *E. coli* via 16S ribosomal RNA gene Sanger sequencing (ABI 3730xl platform, Applied Biosystems, Foster City, CA, USA), the strains were supplemented with glycerol (Sinopharm Chemical Reagent Co., Ltd., Shanghai, China) and stored at −80 °C for subsequent use.

Minimum inhibitory concentrations (MICs) of the isolates against 11 antimicrobial agents were determined using a microbroth dilution method. The assessed antibiotics encompassed polymyxin E, cefotaxime, ceftazidime, tetracycline, tigecycline, amikacin, gentamicin, ciprofloxacin, meropenem, florfenicol, and trimethoprim-sulfamethoxazole (all purchased from Shanghai Aladdin Biochemical Technology Co., Ltd.). *E. coli* ATCC 25922 (American Type Culture Collection, Manassas, VA, USA) was used as a quality control strain, and results were interpreted following the Clinical and Laboratory Standards Institute (CLSI) guidelines.

Genomic DNA was extracted from CTX-M-EC isolates using a TIANamp Bacterial DNA Kit (TianGen Biotech (Beijing) Co., Ltd., Beijing, China), with sequencing performed using the Illumina HiSeq 2500 platform (Illumina, San Diego, CA, USA). The resulting reads were assembled using SPAdes (v3.11) [23], and genomes were annotated using the PATRIC (v3.6.9) platform. Subsequent analyses of sequence types (STs), antimicrobial resistance genes, virulence factors, and plasmid replicon types were performed using the Center for Genomic Epidemiology server (https://cge.food.dtu.dk/ accessed on 15 March 2023). Phylogenetic trees were visualized using iTOL (v6.5.7), and the genetic context of *bla*_CTX-M_ was compared with data from GenBank using EasyFig (v2.2.5) to elucidate its genetic environment.

Using *E. coli* EC600 (HonorGene, Changsha, China) as the recipient strain and CTX-M-EC as the donor strain, both strains were cultured to the logarithmic phase, and their concentrations were adjusted to 0.5 McFarland turbidity (approximately 1–1.5 × 10^8^ CFU/mL). The strains were mixed at a donor-to-recipient volume ratio of 1:3. A 100-μL aliquot of the mixed suspension was spread onto a sterile membrane filter (0.45 μm; BioSharp Biotechnology Co., Ltd., Hefei, China) placed on a Mueller-Hinton agar (Beijing Luqiao Technology Co., Ltd., Beijing, China) plate. After co-incubation at 37 °C for 12 h, the bacterial lawn on the filter was eluted with sterile physiological saline. The eluate was serially diluted and spread onto MacConkey agar plates supplemented with 4 mg/L cefotaxime and 500 mg/L rifampicin (Shanghai Aladdin Biochemical Technology Co., Ltd., Shanghai, China), followed by incubation at 37 °C for 20–24 h. Grayish-white colonies growing on the dual-antibiotic plates were randomly selected, and the presence of the *bla*_CTX-M_ gene was verified by PCR and agarose gel electrophoresis. Negative controls were set by spreading 100 μL of the donor suspension or 100 μL of the recipient suspension alone onto the same dual-antibiotic plates [24].

## 3. Results

### 3.1. CTX-M-EC Separation

From the 278 samples, 121 cefotaxime-resistant strains (an isolation percentage of 43.53%) were isolated, among which 27 strains (9.71%) were CTX-M-EC. Sources of origin were manure samples from piglet pens (5, 10.20%), finishing pens (3, 12.00%), gestation barns (5, 10.00%), farrowing barns (5, 10.00%), boar barn (4, 44.44%), wastewater (3, 16.67%), and flies (2, 13.33%) (Table 1).

### 3.2. Antimicrobial Susceptibility

A comparison of MIC values with respect to CLSI resistance criteria revealed that all CTX-M-EC strains were resistant to cefotaxime, ciprofloxacin, and tetracycline, with more than 90% of strains being resistant to ceftazidime, florfenicol, and trimethoprim-sulfamethoxazole, whereas resistance to gentamicin, polymyxin E, tigecycline, and amikacin was observed in 59.25%, 29.63%, 22.22%, and 14.81% of strains, respectively. All strains were susceptible to meropenem. According to the criteria proposed by Magiorakos et al., MDR is defined as acquired non-susceptibility to at least one agent in three or more antimicrobial classes [25]. All 27 CTX-M-EC isolates were resistant to four or more classes of antimicrobial agents, with 100% multi-drug resistance. Notably, strain P78BP showed resistance to seven classes of antimicrobial agents (excluding meropenem and tigecycline) (Table 2).

### 3.3. ARGs

**Figure 1:**
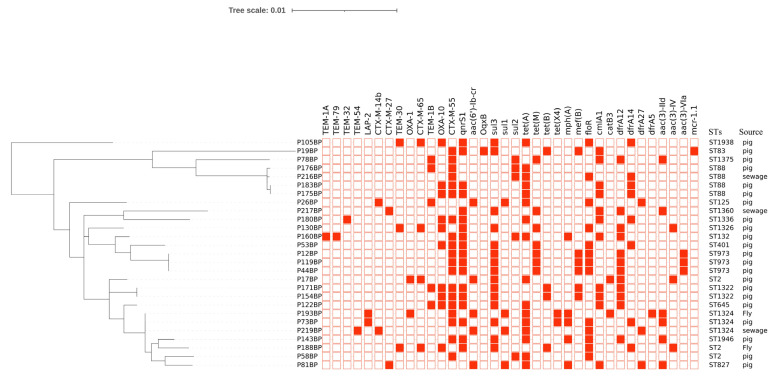
Phylogenetic trees, resistant genes, and STs of 27 strains of CTX-M-EC. **Left**: Phylogenetic tree constructed based on the core genome. Branch lengths represent the nucleotide substitution rate (i.e., evolutionary distance) between isolates or between an isolate and a common ancestor. Shorter branches indicate smaller genomic differences and closer genetic relatedness among isolates, whereas longer branches indicate greater differences and more distant relationships. **Middle**: Heatmap showing resistance genes carried by each isolate, with red squares indicating presence and white squares indicating absence. **Right**: STs and sample sources of the isolates. The scale bar represents 0.01 nucleotide substitutions per site.

Whole-genome sequencing analysis was performed on the 27 isolated MDR *E. coli* strains. Each isolate carried only one *bla*_CTX-M_ variant, and a total of four *bla*_CTX-M_ variants were detected among the 27 strains, belonging to two phylogenetic groups: *bla*_CTX-M-14b_ (*n* = 2, 7.41%), *bla*_CTX-M-27_ (*n* = 2, 7.41%) and *bla*_CTX-M-65_ (*n* = 4, 14.81%) from the CTX-M-9 group, *bla*_CTX-M-55_ (*n* = 19, 70.37%) from the CTX-M-1 group. In addition to *bla*_CTX-M_, other antimicrobial resistance genes (ARGs) conferring resistance to β-lactams, aminoglycosides, amphenicols, tetracyclines, sulfonamides, trimethoprim, macrolides, quinolones, and polypeptides were also present, totaling 36 ARGs. Among these, *qnrS1* (*n* = 18, 66.67%), *tet*(A) (*n* = 16, 59.26%), and *sul3* (*n* = 15, 55.56%) showed relatively high detection rates (Figure 1).

### 3.4. STs

The 27 CTX-M-EC strains were distributed among 17 distinct STs, thereby revealing their genetic diversity. These included four strains of ST88, three strains each of ST2, ST1324, and ST973, two strains of ST1322, and one strain each of the remaining STs (Figure 1).

### 3.5. Virulence Genes

**Figure 2:**
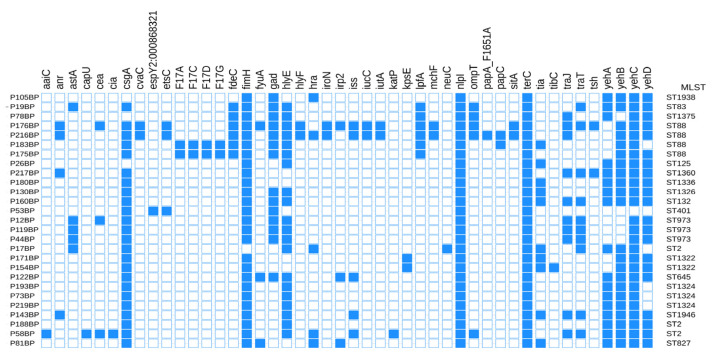
**Left**: Strain names. **Middle**: Heatmap showing virulence genes carried by each isolate, with blue squares indicating presence and white squares indicating absence. **Right**: STs of the isolates.

Analysis of the 27 strains revealed the presence of 46 virulence genes, among which *nlpI* and *terC* were present in all strains, whereas *csgA*, *fimH*, *hlyE*, and *yehB*/*C*/*D* were detected in a majority of strains (Figure 2).

### 3.6. Plasmid Replicons

Plasmid replicon typing analysis of the 27 isolated strains revealed 23 plasmid replicons, among which IncQ1 (14/27), IncFIB(K) (11/27), and IncX1 (13/27) were identified in most isolates, with statistical analysis indicating that almost all positive strains harbored two or more plasmids (Table 3).

### 3.7. Genetic Background

**Figure 3:**
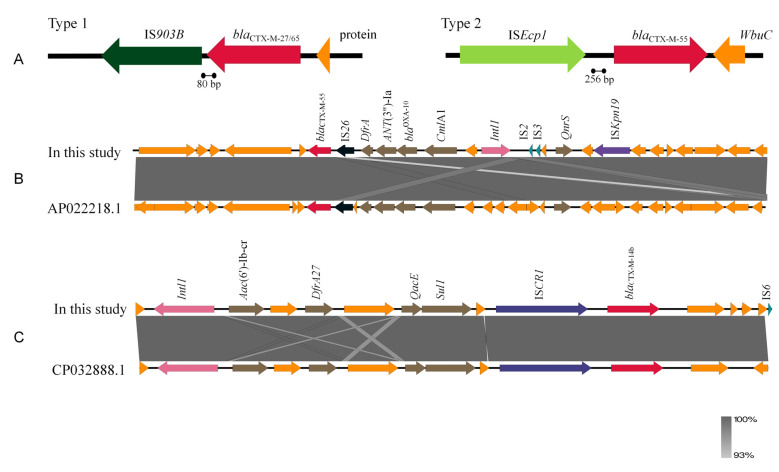
Genetic background of 27 strains of CTX-M-EC. (**A**) From left to right, the genetic environment of *bla*_CTX-M-27_ and *bla*_CTX-M-65_, and the genetic organization of *bla*_CTX-M-55_; (**B**) The genetic environment of the *bla*_CTX-M-55_ strain is highly similar to that of a Japanese sewage treatment plant isolate; (**C**) The genetic environment of *bla*_CTX-M-14b_ is highly similar to that of a human-derived *E. coli* isolate. Arrows represent genes or coding sequences, with direction indicating transcription (5′→3′). Colors denote function: red, *bla*_CTX-M_; brown, other resistance genes; orange, other genes (e.g., WbuC) or hypothetical proteins; other colors, insertion sequences (see labels in the figure for details). Gray shading indicates homology with reference sequences. IS*903B*, IS*Ecp1*, IS*26*, IS*Kpn19*, IS*2*, IS*3*, and IS*CR1* are common insertion sequences; *intI1* is a class 1 integron integrase gene; WbuC is a cupin family metalloprotein (function not fully characterized); the remaining arrows represent resistance genes and their abbreviations, such as *bla*_CTX-M_, *qnrS1*, and *dfrA14*.

Further analysis of the gene environments of the four *bla_CTX-M_* subtypes revealed that both *bla*_CTX-M-27_ and *bla*_CTX-M-65_ had a *bla*_CTX-M_-IS*903B* gene environment, with IS*903B* located 80 bp downstream of *bla_CTX-M_* (Figure 3). The core gene environment of certain *bla*_CTX-M-55_ strains was “IS*Ecp1*-*bla_CTX-M_*,” (Figure 3), whereas the remaining strains harbored multi-drug resistance regions highly similar to those in strains isolated from Japanese sewage treatment plants(GenBank accession number: AP022218.1; Figure 3). The analysis revealed IS*26*-mediated resistance regions for *DfrA14*, *bla*_OXA-10_, and *Cml*A1, and cassette arrays, such as IS*Kpn19*-*qnrS1*, mediated by Class I integrons (*intI1*). The *bla*_CTX-M-14b_ gene environment showed high similarity to that isolated from human-derived *E. coli*(GenBank accession: CP032888.1; Figure 3), both harboring a multi-drug resistance region comprising *sul1*-*qacE*-*DfrA27*-*aac*(6ʹ)-Ib-cr mediated by an *intI1* and insertion sequence common region 1 (collectively termed a composite *intI1*).

### 3.8. Gene Transfer

Following three rounds of conjugation, we established that 96.29% (*n* = 26) of the *bla_CTX-M_* genes had been transferred from the isolates to the recipient *E. coli* EC600 (Table 3).

### 3.9. Transmission Routes of CTX-M-EC

**Figure 4:**
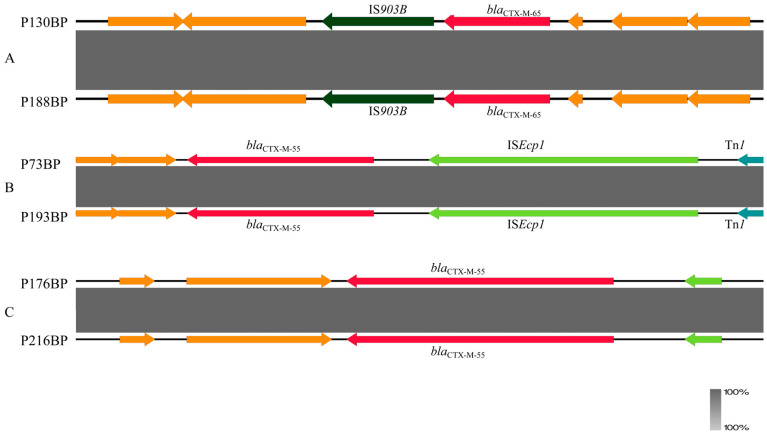
Comparison of gene-environment maps between strains. (**A**) Strains P130BP and P188BP have identical genetic environments. (**B**) The fecal isolate P73BP from the finishing house and the fly isolate P193BP from the farrowing house have identical genetic environments. (**C**) The fecal isolate P176BP from the boar house and the wastewater isolate P216BP have identical genetic environments. The arrows, colors, and shading in this figure have the same meanings as described in Figure 3.

By comparing the phylogenetic tree with the genetic environment maps, it was observed that two distinct transmission routes existed for *bla_CTX-M_*-carrying *E. coli* strains in this pig farm. Strains P130BP and P188BP were placed on distinct branches of the core genome phylogenetic tree and had different STs. Both harbored an identical “*bla*_CTX-M-65_-IS*903B*” drug resistance gene module (Figure 4), which is indicative of ongoing horizontal gene transfer [10]. However, phylogenetic tree analysis revealed that three *E. coli* strains, isolated from farrowing house flies (P193BP), finishing house manure (P73BP), and gestation house wastewater (P219BP), formed a tight phylogenetic cluster with the same ST (Figure 1), indicating that they are members of a recent common transmission clone [26]. Therefore, in this pig farm, CTX-M-EC exhibited dual transmission mechanisms: clonal dissemination and horizontal gene transfer. Meanwhile, comparison of the genetic contexts revealed that strain P73BP from a fecal sample in the finishing barn shared an identical genetic environment with strain P193BP from a fly sample in the farrowing room (Figure 4), while strain P176BP from a fecal sample in the boar room had an identical genetic environment to strain P216BP from a wastewater sample (Figure 4). These findings indicate the presence of transmission chains mediated by flies and wastewater that span production areas within this farm.

## 4. Discussion

### 4.1. Comparison with Previous Studies: Antimicrobial Resistance Rates and Distribution of bla_CTX-M_ Subtypes

The overall isolation rate of cefotaxime-resistant strains in this study was 43.53%, which is similar to that of cefotaxime-resistant strains (46%) from Kim et al. from various farms in South Korea between 2022 and 2023. In that study, *bla*_CTX-M-55_ (59.9%) was also the dominant genotype conferring cefotaxime resistance, a finding highly consistent with the result of the present study (70.37%). They also reported a significantly higher resistance rate on farms with ceftiofur usage, indicating that the widespread use of third-generation cephalosporins is an important driver for the selection and enrichment of resistant strains [8]. In avian-derived *E. coli*, Chen et al. identified 358 CTX-M-positive strains from 1808 chicken-origin *E. coli* isolates collected from 10 provinces in China, among which *bla*_CTX-M-55_ and *bla*_CTX-M-65_ accounted for 42.7% and 25.7%, respectively. This suggests that the prevalence of *bla*_CTX-M-55_ also exists in poultry farming [15], which is consistent with the trend that *bla*_CTX-M-55_ has surpassed *bla*_CTX-M-15_ to become the most common CTX-M subtype in animal-origin *E. coli* in China [15]. The positive rate of CTX-M-EC in this experiment was 9.71%, which was similar to that reported by Masui et al. (9.7%) in a study conducted between 2015 and 2019 using fecal specimens of healthy Japanese individuals [27]. However, it was lower than the rate reported by Wei et al. from cattle feces in different provinces of China (29.6%) [28]. These differences may be attributed to variations in sample types and regional antibiotic usage.

All CTX-M-EC isolates in this study were MDR (100%), which is consistent with trends reported in both Chinese and international studies. Li et al. investigated porcine-origin extraintestinal pathogenic *E. coli* in China and reported an MDR rate of 97% [29], indicating that MDR among swine-origin *E. coli* has become a common phenomenon. All isolates in this study remained 100% susceptible to meropenem, consistent with findings from most global studies on animal-origin *E. coli*, suggesting that carbapenem use in animal husbandry remains effectively controlled [30].

### 4.2. Relationship Between Multilocus Sequence Typing (MLST), Plasmid Types, Insertion Sequences, Virulence Genes, and bla_CTX-M_

In this study, *bla*_CTX-M-55_-carrying strains were distributed among 12 different STs, with ST88 being the predominant ST carrying this gene. *bla*_CTX-M-65_-carrying strains were distributed among three STs, predominantly ST2. Strains carrying *bla*_CTX-M-27_ and *bla*_CTX-M-14b_ were distributed across different STs. Furthermore, strains belonging to ST2 and ST1324 also carried *bla*_CTX-M-55_, indicating that the same ST can carry different CTX-M subtypes by acquiring different plasmids.

In this study, all *bla*_CTX-M-65_-positive strains carried the IncHI2/IncHI2A composite plasmid, whereas it was rarely detected in other strains, suggesting that the IncHI2 plasmid may be a specific vehicle for *bla*_CTX-M-65_. This finding is supported by several studies. For example, in dairy cow-origin *E. coli* from China, *bla*_CTX-M-65_ was located within a Tn3-like composite transposon on an IncHI2 plasmid. Similarly [31], in the swine farm environment in Spain, the genetic context of *bla*_CTX-M-65_ was identified as the conserved IS*903*-*bla*_CTX-M-65_-*fipA* structure on an IncHI2 plasmid [32]. In contrast, the plasmid types carrying *bla*_CTX-M-55_ in this study included more than 20 different types. A genomic epidemiological analysis by Yu et al. involving 1345 CTX-M-carrying plasmids worldwide also confirmed the high diversity of plasmid backbones harboring *bla*_CTX-M-55_ [10]. Therefore, the strong plasmid adaptability of *bla*_CTX-M-55_ may explain why it has become the dominant epidemic subtype in this pig farm and more broadly in animal-origin *E. coli* in China.

The IS*Ecp1*, IS*26*, and IS*903B* elements detected in this study are major mobile elements mediating the horizontal transfer of *bla*_CTX-M_ [33]. Research indicates that IS*Ecp1* insertion sequences are associated with most *bla*_CTX-M_ variants, and the *bla*_CTX-M_ genes associated with this element are typically located within multi-drug resistance regions that frequently carry genes conferring resistance to aminoglycosides, tetracyclines, sulfonamides, or fluoroquinolones [34], which is consistent with the ARG results obtained in the present study.

In this study, all 27 CTX-M-EC isolates carried the *nlpI* and *terC* genes, which are involved in lipoprotein synthesis and tellurite resistance, respectively, and are associated with bacterial stress adaptation. Furthermore, the high detection rates of adhesion-related genes *fimH* (26/27), *csgA* (24/27), and the *yeh* family genes suggest that these isolates possess strong intestinal colonization and biofilm-forming abilities, which may enhance their persistence and transmission potential in the farming environment [35]. Notably, a relatively high proportion of isolates carried *hlyE* and *gad*, genes associated with pathogenicity [36,37], indicating that some CTX-M-EC isolates have potential pathogenic risks.

### 4.3. Public Health Implications and Practical Recommendations for One Health Strategies

The high diversity of MLST indicates that these drug-resistant strains are unlikely to originate from the dissemination of a single clonal lineage but rather are likely transmitted through horizontal gene transfer. Although the overall diversity was high, a few STs, such as ST88 (4 isolates), ST2 (3 isolates), ST1324 (3 isolates), and ST973 (3 isolates), were isolated multiple times, suggesting that these clones may possess enhanced adaptability and transmission capacity within the farming environment. ST88 *E. coli* can carry multiple resistance genes and has been isolated from human clinical samples [38]. In this study, ST88 clone strains were present in both pig feces (P176BP) and wastewater (P216BP), indicating that this clone may spread through animals and the environment, posing a potential animal–environment–human transmission risk. Furthermore, ST88 strains carried more virulence genes than other STs. Among them, P176BP and P216BP carried more than 27 virulence genes (including the iron uptake-related genes *iucC*/*iutA* and *iroN*, as well as the adhesins *fimH* and *papC*), indicating that the ST88 clone may represent an epidemic lineage with high virulence potential in the pig farm environment. Therefore, surveillance of high-risk clones such as ST88 should be incorporated into the antimicrobial resistance control system in livestock farms. This also implies that controlling antimicrobial resistance must shift from single-animal treatment to integrated management, elevating pest control and wastewater disinfection to core biosecurity strategies.

The *bla*_CTX-M_ genes are frequently located on conjugative plasmids, among which incompatibility (Inc) group F plasmids are the most common [10], which is consistent with the finding in this study that 92.59% (*n* = 25) of the strains carried IncF plasmids. This study reported that 96.29% of the isolated strains harbored *bla*_CTX-M_ genes with transferability. Therefore, it is necessary to strengthen surveillance of such strains to prevent their entry into the human food chain through contaminated meat products or environmental discharge, thereby threatening public health safety [39].

Previously, we have also observed that all multi-drug-resistant strains harboring mobilized colistin resistance gene 1 (*mcr*-1) at this pig farm simultaneously carried at least one β-lactam resistance gene, on the basis of which, we hypothesized that the prevalence of ESBLs may have generated selective pressure, facilitating the colonization and spread of strains carrying *mcr*-1 [40]. Our findings in this study provide strong environmental evidence supporting this hypothesis. Controlling the prevalence of ESBL genes, such as *bla*_CTX-M_, on farms is essential for addressing β-lactam antibiotic resistance and preserving the efficacy of polymyxin, a key last-resort antibiotic. Consequently, antimicrobial resistance management on livestock farms should focus not solely on individual resistance genes but adopt a collaborative “One Health” approach.

Based on the findings of this study, the following integrated measures are recommended to curb the spread of CTX-M-EC in pig farms: (i) immediate discontinuation of the prophylactic use of β-lactam antibiotics and reserving their administration for treatment guided by antimicrobial susceptibility testing; (ii) application of high-temperature composting or anaerobic fermentation to feces, and disinfecting wastewater by UV or chlorine treatment before discharge; (iii) installation of insect-proof nets and eliminating standing water to reduce vector-borne transmission by flies; and (iv) routine monitoring of pig feces for the presence of *bla*_CTX-M_ and *mcr*-1 genes, as well as high-risk ST88 strains. The synergistic implementation of these measures can effectively interrupt the transmission chain of resistance genes within the pig farm ecosystem.

## 5. Conclusions

Our survey of a pig farm in China revealed a widespread dissemination of the *bla*_CTX-M_ gene, with all bacterial carriers being characterized by multi-drug resistance, thereby highlighting the need to strengthen on-farm surveillance. Furthermore, the detection of resistant bacteria in wastewater and insect environmental samples confirms that drug-resistant bacteria can spread through the environment. More alarmingly, 96.29% of isolates harbored *bla*_CTX-M_ with transferable capabilities. Therefore, enhanced manure management is essential to interrupt environmental transmission chains, thereby reducing the spread of resistant bacteria and safeguarding public health.

## Figures and Tables

**Table 1:** Sampling overview and distribution of cefotaxime-resistant *E. coli* and CTX-M-EC.

Sample Type	Sampling Site	Number of Samples	Number of Cefotaxime-Resistant *E. coli*	Number of CTX-M-EC
Manure	Piglet house	49	25 (51.02%)	5 (10.20%)
	Gestation house	50	23 (46.00%)	5 (10.00%)
	Farrowing house	50	34 (68.00%)	5 (10.00%)
	Finishing house	25	14 (56.00%)	3 (12.00%)
	Boar house	9	6 (66.67%)	4 (44.44%)
Wastewater	Piglet house	4	0	0
	Gestation house	4	3 (75.00%)	2 (50.00%)
	Farrowing house	4	2 (50.00%)	0
	Finishing house	4	1 (25.00%)	0
	Boar house	2	1 (50.00%)	1 (50.00%)
Drinking water	Piglet house	4	0	0
	Gestation house	4	0	0
	Farrowing house	4	0	0
	Finishing house	4	2 (50.00%)	0
	Boar house	2	0	0
Fly	Piglet house	3	1 (33.33%)	0
	Gestation house	3	2 (66.67%)	1 (33.33%)
	Farrowing house	3	2 (66.67%)	1 (33.33%)
	Finishing house	3	2 (66.67%)	0
	Boar house	3	3 (100.00%)	0
Soil	Surroundings of pig houses	16	0	0
Passage	Personnel disinfection passage	5	0	0
	Vehicle disinfection passage	3	0	0
	Quarantine area	5	0	0
	Pig driving passage	15	0	0
Total		278	121 (43.53%)	27 (9.71%)

**Table 2:** Drug resistance rate and MIC (mg/L) of 27 strains of CTX-M-EC.

Strain	*bla*_CTX-M_ Variant	GEN	AMK	CAZ	CTX	MEM	FFC	CIP	TET	TGC	COL	TMP-SMX
P12BP	*bla*_CTX-M-55_	>128	1	>128	>128	0.125	>128	>128	>128	0.25	2	>128
P17BP	*bla*_CTX-M-65_	>128	8	32	>128	0.5	>128	64	>128	0.5	2	>128
P19BP	*bla*_CTX-M-55_	2	4	>128	>128	0.25	>128	>128	>128	0.125	16	>128
P26BP	*bla*_CTX-M-14b_	2	64	128	>128	0.25	>128	16	>128	0.5	128	>128
P44BP	*bla*_CTX-M-55_	>128	8	>128	>128	0.125	>128	128	>128	0.25	2	>128
P53BP	*bla*_CTX-M-55_	1	64	>128	>128	0.125	>128	32	>128	0.5	2	>128
P58BP	*bla*_CTX-M-55_	2	8	64	>128	0.25	>128	1	>128	0.5	2	16
P73BP	*bla*_CTX-M-55_	>128	8	>128	>128	0.125	>128	>128	>128	8	2	>128
P78BP	*bla*_CTX-M-55_	>128	8	>128	>128	0.125	>128	>128	>128	>128	4	>128
P81BP	*bla*_CTX-M-27_	>128	4	>128	>128	0.125	>128	32	>128	0.25	2	>128
P105BP	*bla*_CTX-M-65_	1	2	64	128	0.125	>128	>128	>128	0.25	1	>128
P119BP	*bla*_CTX-M-55_	>128	8	>128	>128	0.25	>128	32	>128	0.5	2	>128
P122BP	*bla*_CTX-M-55_	0.5	4	>128	16	0.125	>128	4	128	0.25	4	>128
P130BP	*bla*_CTX-M-65_	>128	4	>128	128	0.125	>128	>128	64	0.125	8	>128
P143BP	*bla*_CTX-M-55_	>128	8	>128	>128	0.125	>128	>128	>128	>128	2	>128
P154BP	*bla*_CTX-M-55_	16	8	128	64	0.125	>128	>128	>128	0.125	1	8
P160BP	*bla*_CTX-M-55_	2	64	>128	>128	0.125	>128	>128	>128	0.5	2	>128
P171BP	*bla*_CTX-M-55_	1	4	32	>128	0.125	>128	16	>128	0.125	4	>128
P175BP	*bla*_CTX-M-55_	2	8	>128	>128	0.25	32	>128	>128	>128	2	>128
P176BP	*bla*_CTX-M-55_	16	2	>128	>128	0.25	1	64	>128	0.25	2	>128
P180BP	*bla*_CTX-M-55_	1	8	>128	>128	0.125	>128	4	>128	0.25	4	>128
P183BP	*bla*_CTX-M-55_	2	4	>128	>128	0.125	16	>128	64	0.25	2	>128
P188BP	*bla*_CTX-M-65_	>128	8	4	>128	0.125	64	>128	>128	0.125	1	>128
P193BP	*bla*_CTX-M-55_	>128	64	128	>128	0.125	>128	4	>128	8	2	>128
P216BP	*bla*_CTX-M-55_	>128	8	>128	>128	0.125	>128	>128	>128	0.25	8	>128
P217BP	*bla*_CTX-M-27_	>128	1	32	32	0.125	>128	4	64	0.25	2	>128
P219BP	*bla*_CTX-M-14b_	64	4	4	64	0.25	>128	1	>128	8	2	>128
resistance rate(%)	-	59.25	14.81	92.59	100.00	0	92.59	100.00	100.00	22.22	29.63	92.59

GEN, Gentamicin; AMK, Amikacin; CAZ, Ceftazidime; CTX, Cefotaxime; MEM, Meropenem; FFC, Florfenicol; CIP, Ciprofloxacin; TET, Tetracycline; TGC, Tigecycline; COL, Colistin; TMP-SMX, trimethoprim-sulfamethoxazole.

**Table 3:** Plasmid conjugation and plasmid replicon of 27 strains of CTX-M-EC.

Strain	Transferable (+/−)	Plasmid Types
P12BP	+	IncFIB(K), IncFII(pHN7A8), IncQ1, p0111
P17BP	+	Col156, IncFII, IncHI2, IncHI2A, IncY
P19BP	+	IncFIA(HI1), IncFIB(K), IncFII, IncI1-I(Alpha), IncX4
P26BP	−	IncFIB(K), IncQ1
P44BP	+	IncFIB(K), IncFII(pHN7A8), IncQ1, p0111
P53BP	+	IncQ1, IncX1, IncY
P58BP	+	Col(KPHS6), IncFII, IncFII(pHN7A8)
P73BP	+	IncFIA(HI1), IncFIB(K), IncHI2, IncHI2A, IncQ1, IncX1
P78BP	+	IncFIB(AP001918), IncFII(pHN7A8), Col(pHAD28)
P81BP	+	IncFIA(HI1), IncHI1A, IncHI1B(R27), IncQ1, IncX1
P105BP	+	IncFIA(HI1), IncFIB(K), IncHI2, IncHI2A, IncQ1, Col(pHAD28)
P119BP	+	IncFIB(K), IncFII(pHN7A8), p0111
P122BP	+	IncFIA(HI1), IncFIB(K), IncQ1, IncX1, p0111, Col(pHAD28)
P130BP	+	IncHI2, IncHI2A, IncQ1
P143BP	+	IncFIB(AP001918), IncI1-I(Alpha), IncR, IncX1
P154BP	+	IncX1, IncFIB(pHCM2)
P160BP	+	IncFIB(AP001918), IncFIB(K), IncFII(pHN7A8), IncQ1, IncR, IncX1
P171BP	+	IncQ1, IncX1, IncFII(pHCM2)
P175BP	+	IncX1, IncFII
P176BP	+	IncFIB(AP001918), IncFIC(FII), IncFII(pHN7A8), IncN
P180BP	+	IncX1
P183BP	+	IncX1, IncHI2, IncFIA(HI1), IncHI2A
P188BP	+	IncFIB(AP001918), IncHI2, IncHI2A, IncQ1
P193BP	+	IncFIA(HI1), IncFIB(K), IncHI2, IncHI2A, IncQ1, IncX1
P216BP	+	IncFIB(AP001918), IncFIC(FII), IncFII(pHN7A8), IncN
P217BP	+	IncFIB(AP001918), p0111, IncFIC(FII)
P219BP	+	IncFIB(K), IncQ1, IncX1

+, positive (successful transfer); –, negative (failed transfer).

## Data Availability

Genome assembly of 27 *Escherichia coli* strains in this study was deposited in GenBank under the BioProject accession: PRJNA1394679.

## Abbreviations

The following abbreviations are used in this manuscript:

AMR	antimicrobial resistance
*bla_CTX-M_*	β-lactamase, cefotaxime-hydrolyzing, Munich
*E. coli*	*Escherichia coli*
ESBLs	extended-spectrum beta-lactamases
ARG	antibiotic resistance gene
CTX-M-EC	*bla_CTX-M_*-positive *E. coli*
PCR	polymerase chain reaction
MIC	minimum inhibitory concentration
STs	sequence types
MDR	multi-drug resistance
